# Symptom Burden and Palliative Referral Disparities in an Ambulatory South Texas Cancer Center

**DOI:** 10.3389/fonc.2018.00443

**Published:** 2018-10-15

**Authors:** Sherri Rauenzahn Cervantez, Laura L. Tenner, Susanne Schmidt, Ifeoma O. Aduba, Jessica T. Jones, Nazneen Ali, Savitri Singh-Carlson

**Affiliations:** ^1^Medical Oncology, The University of Texas Health Science Center at San Antonio, San Antonio, TX, United States; ^2^Department of Epidemiology and Biostatistics, The University of Texas Health Science Center at San Antonio, San Antonio, TX, United States; ^3^School of Nursing, San Diego State University, San Diego, CA, United States

**Keywords:** palliative, symptom assessment, disparities, Hispanic, cancer, ESAS, safety-net

## Abstract

**Background:** The American Society of Clinical Oncology's recommendation for “dedicated palliative care services, early in the disease course, concurrent with active treatment” for cancer patients is a challenge for cancer centers to accommodate. Despite demonstrated benefits of concurrent care, disparities among socioeconomic and ethnic groups in access to supportive care services have been described. The aim of this project was to evaluate: (a) how insurance coverage and ethnicity impact patient symptom burden and, (b) how those factors influence palliative access for patients at a South Texas NCI-designated cancer center.

**Methods:** During a 5-month prospective period, 604 patients from five ambulatory oncology clinics completed the 10 question Edmonton Symptom Assessment Scale (ESAS) surveys during their clinic visit. Patient demographics, ESAS scores, palliative referral decisions, and time to palliative encounters were collected. We compared symptom burden and time to consult based on ethnicity and insurance status (insured = Group A; under-insured and safety net = Group B).

**Results:** The mean ESAS score for all patients at the initial visit was 19.9 (*SD* = 18.1). Safety net patients were significantly more likely to be Hispanic, younger in age, and have an underlying GI malignancy in comparison to insured patients; however, the symptom severity was similar between groups with over 40% of individuals reporting at least one severe symptom. Twenty-one referrals were made to palliative care. On average, Group B had 33.3 days longer wait times until their first potential visit (*p* < 0.01) when compared to Group A. Time to actual visit was on average 57.6 days longer for patients in Group B compared to patients in Group A (*p* = 0.01), averaging at 73.8 days for safety net patients.

**Conclusions:** This project highlights the high symptom burden of oncology patients and disparities in access to services based on insurance coverage. This investigation revealed a 4-fold increase in the time to the first scheduled palliative care visit based on whether patients were insured vs. under-insured. While this study is limited by a small sample size, data suggest that under-insured oncology patients may have significant barriers to palliative care services, which may influence their cancer care quality.

## Introduction

Palliative medicine is a multidisciplinary subspecialty dedicated to improving quality of life (QOL) by alleviating physical, emotional, psychological, and spiritual suffering for patients throughout their disease course (1, 2). Over the last decade, palliative medicine in oncology has increasingly played a larger role in high quality patient care (3, 4). A large part of this change can be attributed to the growing clinical data supporting the integration of palliative care into routine oncological care with demonstrated benefits such as improving QOL, treatment decision making, care satisfaction, healthcare utilization, and overall survivability for cancer patients (4–6). While the collaboration of palliative and oncology care is beneficial, access to timely palliative care services remains an issue for some cancer patients (7–9). One area of concern is the possible disparities in certain ethnic and socioeconomic groups, specifically the Hispanic population and low income groups (7, 9–11).

The American Cancer Society recently reported that ~1 in 3 Hispanics will be diagnosed with cancer in their lifetime, with 1 in 5 men and 1 in 6 women dying from cancer, making cancer the leading cause of death among Hispanics (12, 13). Moreover, Hispanic lung, head and neck, or gastrointestinal cancer patients account for ~52 and 40% of cancer mortality among male and female cancer patients, respectively (12).

Cancer is the cause of 1 out of every 4 deaths in the United States (13) and is the leading cause of death in 22 states (14). One out of every three working-age cancer patient struggles with debt from the high cost of cancer treatments (15). One study observed that cancer patients who had limited financial resources, reported higher pain and poorer quality of life (16). Texas has one of the highest uninsured rates in the country with over 4.5 million citizens without health coverage or 16.6% in 2016 (17, 18), which is double the national average of 8.6% (19).

Our South Texas safety-net National Cancer Institute (NCI)-designated cancer center plays an active role in reducing distress, burden, and morbidity of cancer for patients and their families in a predominantly Hispanic, low socioeconomic population. Identifying symptom trends for patients that are based on social/cultural and socioeconomic factors may better inform our center's practices and programs, as well as provide important information to other cancer centers across the nation as the Hispanic population continues to grow nationally.

In light of our South Texas location that serves a diverse population, the aim of this pilot project was: (a) To evaluate how insurance coverage and ethnicity impact distribution of symptom burden in ambulatory oncology across different primary cancers, specifically lung, head and neck, gastrointestinal (GI) and breast malignancies, and (b) To evaluate how these factors influence palliative access for patients in a South Texas NCI-designated cancer center.

## Materials and methods

### Cancer center and clinics

Our cancer center is one of four NCI-designated cancer centers in Texas and serves a large, predominantly Hispanic South Texas population. The cancer center partners with the University Health System and county hospital, thereby serving as the only safety-net cancer center in South Texas. As a safety-net cancer center, it provides access to cancer care for indigent and uninsured patients in Bexar County. In 2016, the cancer center saw more than 3,300 newly diagnosed patients with cancer in disease site specific clinics (20).

During a 5-month prospective observational period, socio-demographic information, Edmonton Symptom Assessment Scale (ESAS) scores and palliative care referral information were collected for 607 patients from the thoracic, head and neck, GI, and breast ambulatory oncology clinics at one South Texas Cancer Clinic. Three patients had incomplete insurance coverage information and were therefore excluded from the analysis, resulting in 604 patients with completed ESAS and socio-demographic forms. The time from consultation to first palliative visit was captured for referred patients. This single center prospective study was approved by the Institutional Review Board at University of Texas Health Science Center San Antonio (UTHSCSA).

### Palliative care referrals

The referral location of palliative care services within the University of Texas Medicine System was dependent on the type of insurance coverage. Insured patients were eligible to receive palliative care through the University of Texas Medicine System Palliative Care and Geriatrics Family Medicine clinic (Group A) while under-insured patients and patients covered by safety-net programs could receive their palliative care at the University Health System through the Internal Medicine Palliative Care Clinic (Group B). Twenty-one unique patients were referred at their first visit during the 5-month study period.

### Data collection and instruments

The Edmonton Symptom Assessment Scale (ESAS) (see Appendix 1 in Supplementary Material) is a standardized tool validated in multiple languages (including English and Spanish) which assesses symptoms for patients with cancer (21–30). The ESAS tool consists of 10 items that capture general symptom burden (e.g., pain, fatigue, nausea, depression, etc.) scored on a scale from 0 (no symptom) to 10 (worst possible symptom). Data show that scores of 7 or higher indicate severe symptom burden and that a change by plus or minus 1 point represents a clinically significant symptom burden change (31–35). A total symptom burden assessment can be achieved by adding up the individual scores to the 10 symptoms assessed resulting in a total ESAS score between 0 and 100. In addition, the symptoms assessed can be organized to produce physical and emotional sub-scores (36). Adding the scores for pain, fatigue, nausea, drowsiness, dyspnea, loss of appetite, and wellbeing represent the physical sub-score (range 0–70). Summing both anxiety and depression yields the emotional or psychological sub-scale (range 0–20).

The ESAS questionnaire was administered by trained medical assistants at each clinic visit, regardless of disease status and prior responses or referrals. The completed ESAS forms were reviewed by the physician provider during each visit to decide if a palliative referral was appropriate based on patient-reported symptom burden. Study data, including patient demographics, cancer type, ESAS scores, palliative care referral, and insurance type were collected and managed using REDCap (Research Electronic Data Capture) tools hosted at UTHSCSA (37).

### Data analysis

**Figure 1:**
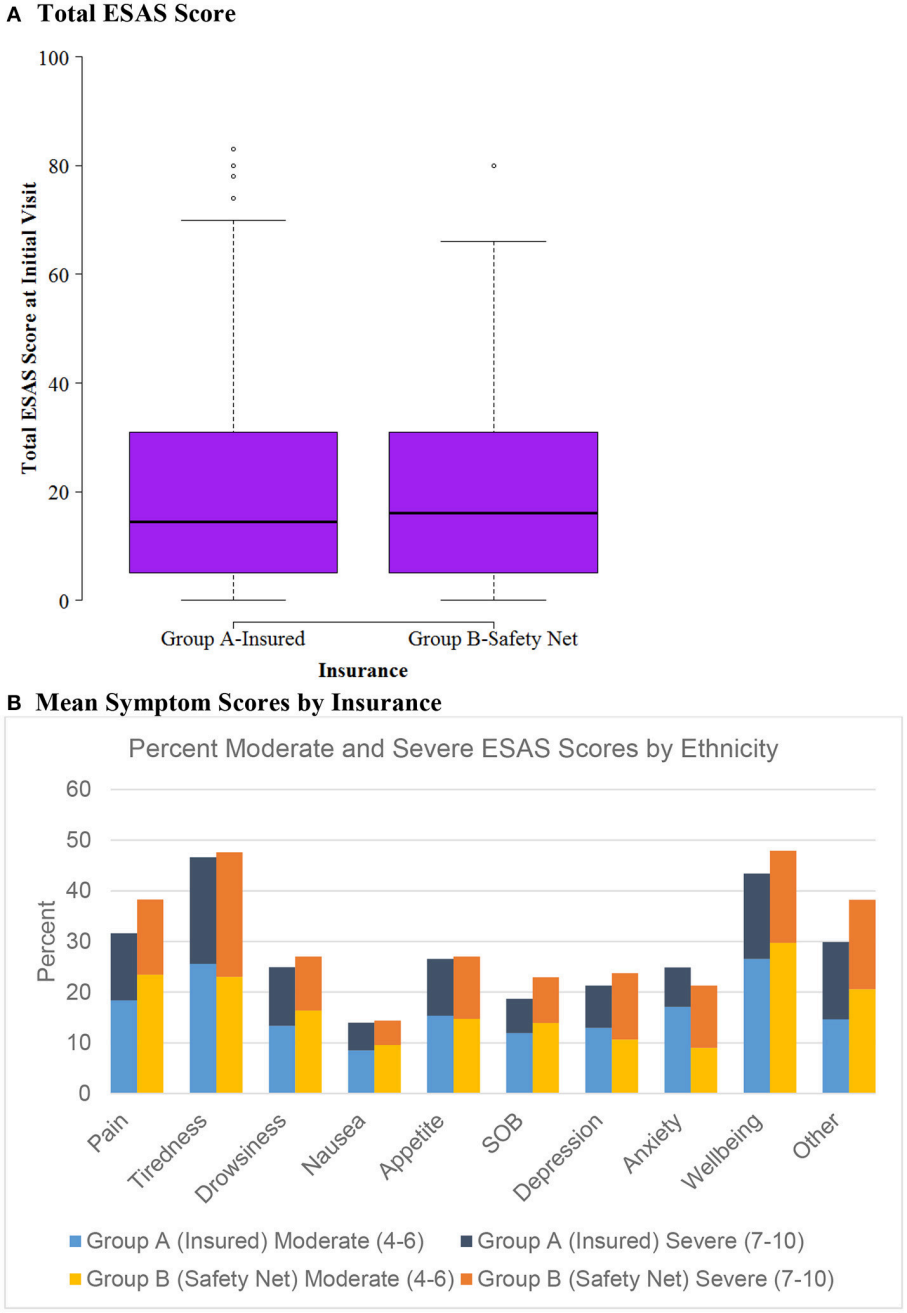
ESAS score by insurance status (*n* = 604). **(A)** Total ESAS score. **(B)** Mean symptom scores by insurance.

**Figure 2:**
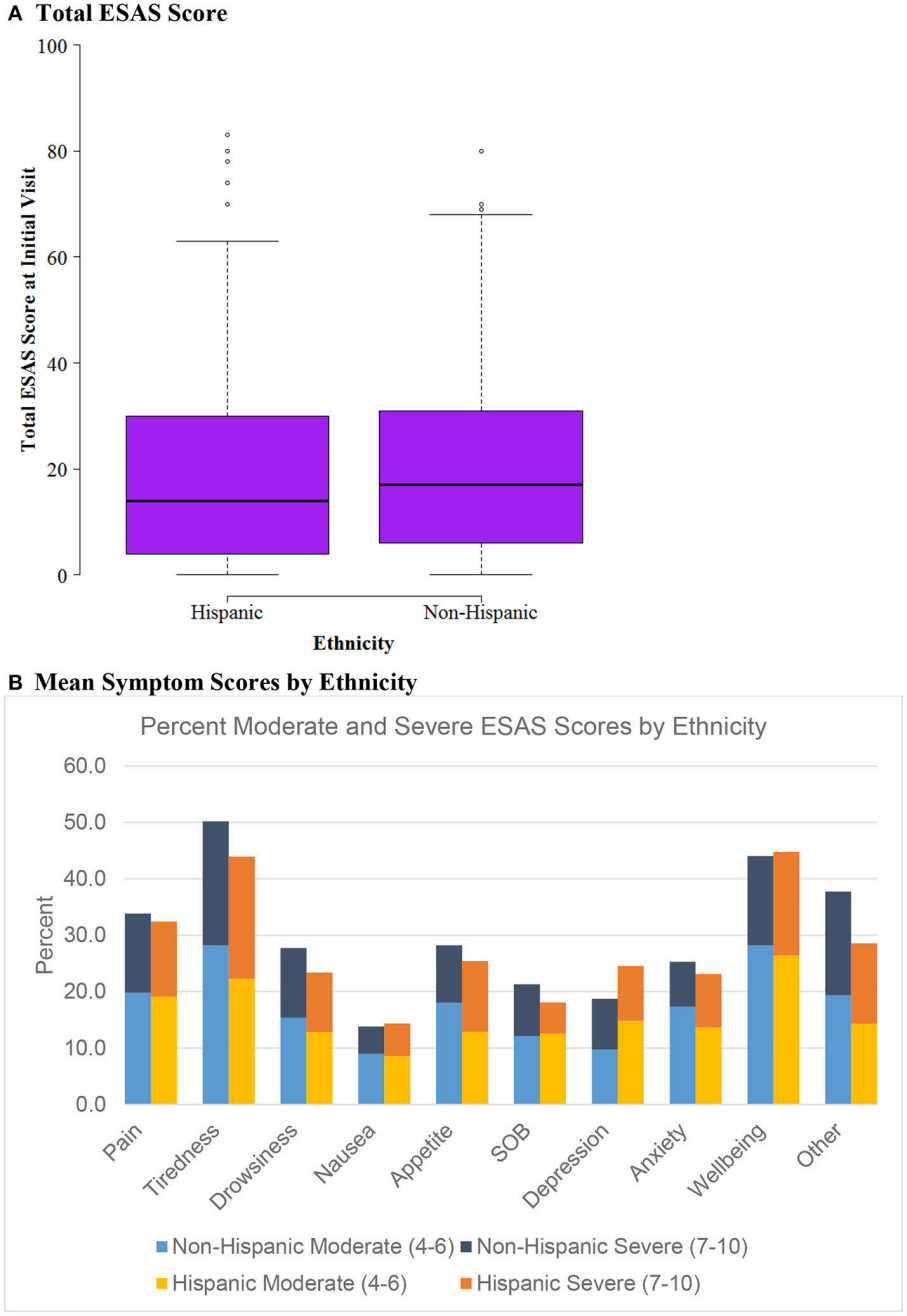
ESAS score by Ethnicity (*n* = 604). **(A)** Total ESAS score. **(B)** Mean symptom scores by ethnicity.

**Figure 3:**
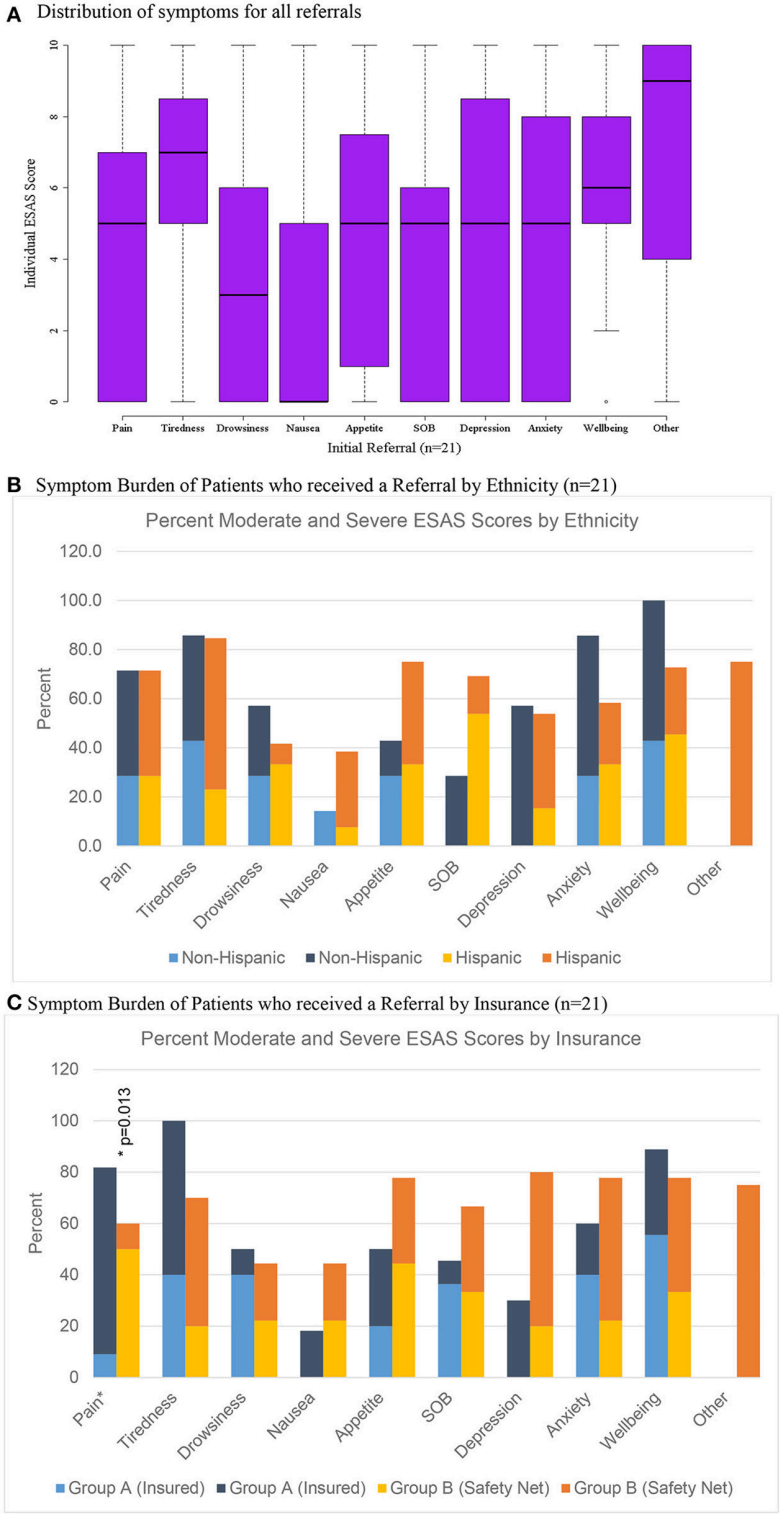
Distribution of scores for patients referred to palliative care (*n* = 21). **(A)** Distribution of symptoms for all referrals. **(B)** Symptom burden of patients who received a referral by ethnicity (*n* = 21). **(C)** Symptom burden of patients who received a referral by insurance (*n* = 21).

Patient socio-demographic data, ESAS scores, and referral information were analyzed using SAS 9.4 (Cary, NC). R was used for graphing boxplots (Figures 1, 2, 3A) (38). We used univariate descriptive statistics to describe our patient population and symptom burden at initial and follow-up visit (Tables 1, 2). Further, we examined changes in ESAS physical and emotional sub-scores at initial visit and follow-up. We used paired *t*-tests to test these differences (Table 3). Then we used bivariate descriptive statistics to compare patient characteristics by insurance coverage (Table 4) as well as characteristics of those referred to different palliative care locations based on patient insurance coverage (Table 5). We also examined differences in total and individual ESAS scores for patients by insurance coverage (Figure 1) and ethnicity (Figure 2). Lastly, we present symptom burden distribution for referrals to palliative care by ethnicity and insurance (Figures 3B,C).

**Table 1:** Demographic and cancer characteristics.

	**Initial visit % (*n*)**	**1st Follow-up Visit % (*n*)**	*p*-value^*^
Number of Patients	604	240	–
Age in years, mean (Std, range)	59.9 (11.9; 27.0–95.0)	59.7 (11.3; 27.0–95.0)	0.67
Sex			0.08
Male	41.9 (253)	46.3 (111)	
Female	58.1 (351)	53.8 (129)	
Race/Ethnicity			0.80
Hispanic	53.9 (326)	52.5 (126)	
Non-Hispanic	44.2 (267)	45.4 (109)	
White
Other/Unknown	1.8 (11)	2.1 (5)	
Insurance Type			<0.01
Carelink	21.2 (128)	27.5 (66)	
Other	78.8 (476)	72.5 (174)	
Cancer Diagnosis			<0.01
Breast	21.9 (132)	12.1 (29)	
Gastrointestinal	53.8 (325)	63.3 (152)	
Lung	15.6 (94)	17.5 (42)	
Head and neck	5.6 (34)	5.4 (13)	
Other	3.2 (19)	1.7 (4)	

* *p-value from Chi-Squared test comparing those with (n = 240) and without (n = 364) a 1st follow-up visit*.

**Table 2:** Symptom burden and referral to palliative care.

	**Initial visit**	**1st Follow-up % (*n*)**
Number of patients	604	240
Symptom Burden		
Total ESAS Score, mean (Std; range)	19.9 (18.1; 0–83)	20.6 (18.2; 0–83)
Emotional Subscore, mean (Std; range)	3.6 (4.9; 0–20)	3.8 (5.1; 0–20)
Physical Subscore, mean (Std; range)	15.4 (13.8; 0–61)	15.7 (13.8; 0–69)
Patients with at least 1 ESAS score >=7, % (*n*)	41.1% (248)	40.8 (98)
Referral to Palliative Care, % (*n*)		
Received referral	3.5% (21)	1.7% (4)
Did not receive referral	96.5% (583)	98.4 (236)
Total ESAS score by referral status, mean (Std; range)		
Received referral	39.1 (19.1; 13–80)	33.8 (12.2; 22–49)
Did not receive referral	19.1 (17.6; 0–83)	20.4 (18.1; 0–83)

**Table 3:** Change in ESAS scores for patients with at least one follow-up visit (*n* = 240).

	**ESAS, Mean (SD)**			**% Change from initial visit (%)**	***P*-value**
	**Initial visit**	**First follow-up visit**	**Change**		
Physical (0–70)	17.1 (14.6)	15.7 (13.8)	−1.3 (12.9)	−7.6	0.13
Emotional (0–20)	3.9 (5.2)	3.8 (5.1)	−0.00 (4.7)	−0.3	0.99
Total (0–90)	20.8 (18.1)	19.5 (17.2)	−1.3 (15.4)	−6.2	0.19
*Patients with referral (n = 21)*					
Physical (0–70)	27.6 (14.5)	25.6 (14.4)	−2.0 (11.8)	−7.3	0.51
Emotional (0–20)	8.4 (7.4)	5.0 (6.3)	−3.3 (6.9)	−40.0	0.10
Total (0–90)	35.4 (18.3)	30.3 (17.4)	−5.1 (17.5)	−14.5	0.26

*p-value from paired t-tests*.

**Table 4:** Demographic and cancer characteristics by insurance (*n* = 604).

	**Group A: Insured (*N* = 476)**	**Group B: Safety Net Program (*N* = 128)**	***p*-value**
Mean Age in years (Std; range)	60.9 (12.5; 27–95)	56.3 (8.7; 31–89)	<0.01
Male, no. (%)	198 (40.6%)	60 (46.9%)	0.20
Race/Ethnicity			<0.01
Hispanic	240 (50.4%)	86 (67.2%)	
Non-Hispanic White	228 (47.9%)	39 (30.5%)	
Other/Unknown	8 (1.7%)	3 (2.3%)	
Cancer Diagnosis, no. (%)			<0.01
Breast	119 (25%)	13 (10.2%)	
Gastrointestinal	231 (48.5%)	94 (73.4%)	
Lung	80 (16.8%)	14 (10.9%)	
Head and Neck	28 (5.9%)	6 (4.7%)	
Other	18 (3.8%)	1 (0.8%)	
Mean Total ESAS score (Std; range 0–100)	19.5 (17.7; 0–83)	21.2 (19.5; 0–80)	0.35
Patients rating a severe symptom of 7 or greater at initial visit, no. (%)	193 (40.6%)	55 (43.0%)	

**Table 5:** Referral characteristics of palliative services.

**Location of services**	**UT-Med (Group A)**	**UHS (Group B)**	***p*-value**
Number of patients referred, (%)	11 (2.3%)	10 (7.8%)	–
Percent of referred patients who are Hispanic, % (*n*)	54.6% (6)	80.0% (8)	0.22
Mean ESAS score at time of referral (Std; range)	34.9 (15.4; 19–61)	43.6 (22.3; 13–80)	0.11
Mean days between consult and first offered visit (Std; range; *n*)	10.6 (8.1; 2–22; 11)	43.9 (15.6; 19–65; 8)	< 0.01
Mean days between consult and actual 1st visit (std;range; *n*)	16.2 (13.0; 2–41; 10)	73.8 (57.7; 19–155; 4)	0.02

## Results

### Characteristics of total patient population

A total of 604 patients completed the ESAS on at least one clinical visit. Our initial patient population had a mean age of 59.9 years with 58.1% female, 53.9% Hispanic patients, 21.2% of patients covered by a safety net payment program and were predominately gastrointestinal cancer (53.8%) and breast cancer (21.9%) (Table 1). The mean total ESAS score [19.9; standard deviation (Std) 18.1] and the percentage of patients reporting severe symptoms (≥7) for any category (41.4%; *n* = 248) were similar from initial visit to first follow-up encounter (Table 2). The demographics for follow-up visits were similar to our initial population with 240 patients returning for at least one visit and 430 total follow-up forms collected.

Of the 240 patients with at least one follow-up encounter during the 5 months study period, the total ESAS score was 20.6 (Std = 18.2) with physical and emotional sub-scores of 17.1 (Std = 14.6) and 3.9 (Std = 5.2), respectively. There were no statistically significant differences between the initial visit and follow-up visit sub-scores (Table 3).

### Characteristics by insurance status and ethnicity

Patients were categorized into either Group A, insured, (*n* = 476) or Group B, safety net, (*n* = 128) cohorts. Safety net patients (Group B) were statistically significantly more likely to be younger, Hispanic, and have an underlying GI malignancy in comparison to the insured patients (group A) (Table 4). Symptom burden in terms of mean total ESAS and rated symptom severity were similar between groups (Table 4). Furthermore, each individual symptom assessment score in the two groups was evaluated by separating the moderate scores from the severe scores and showed no significant differences in individual symptom burden between insurance status groups (Figure 1).

Figure 2 evaluates ESAS score differences by ethnicity, evaluating Hispanic vs. Non-Hispanic cohorts. Similarly to the insurance status groups, there was no difference between mean ESAS scores or distribution and no significant differences in symptom burden between Hispanics vs. Non-Hispanics.

### Characteristics of patients referred to palliative care

Only 3.5% (*n* = 21) of all patients were initially referred to palliative care and 1.7% (*n* = 4) of patients were referred on follow-up visits (Table 2). Patients who were referred to palliative services on initial completion of the ESAS form had a mean score of 39.1 (Std = 19.1) compared to non-referred patients with a mean score of 19.1 (Table 2). Tiredness and “other symptom” were the most commonly reported severe symptoms for patients referred to palliative services while all remaining symptom categories had broader distributions in the mild or moderate score ranges (Figure 3A). Referred patients had more than double the emotional sub-score compared to non-referred patient and had a 40% improvement in emotional sub-scores at first follow-up (Table 3).

Of the 21 patients referred to palliative care after their initial visit, 11 patients were eligible to receive care through the University of Texas (UT) Medicine System (Group A) and 10 patients qualified for care at University System, which provides services to under-insured and safety net patients (Group B) (Table 5). There was no significant differences in symptom distribution based on Hispanic ethnicity or insurance (Group A or B) (Figures 3B,C). However, insured patients with a referral (Group A) were statistically more likely to rate pain as more severe in this limited sample size (Figure 3C).

**Figure 4:**
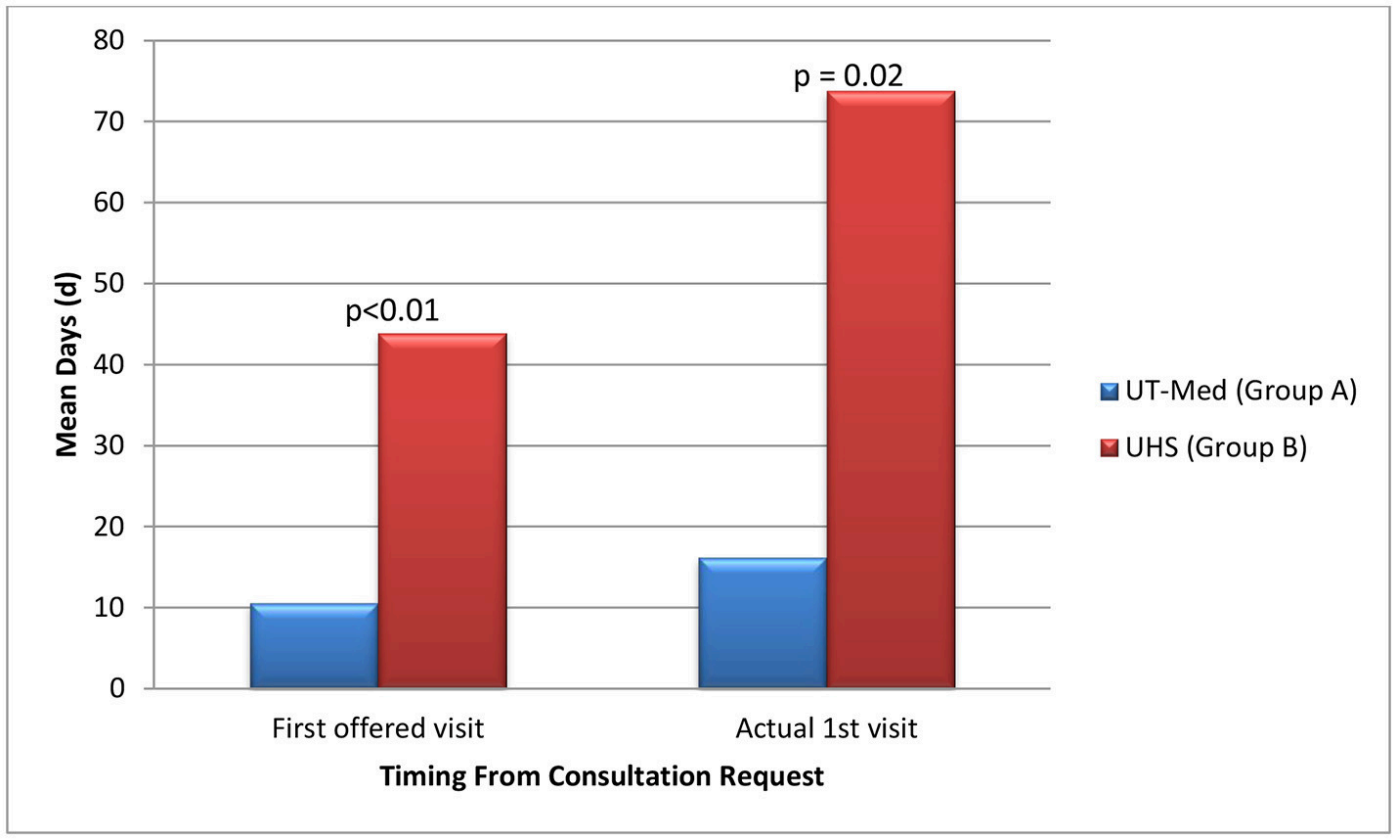
Time from referral to palliative service.

On average, patients seeking care at the safety net facility (Group B) had 33.3 days longer wait times until their first potential visit (*p* < 0.01) compared to patients eligible for UT Medicine (Group A) (Table 5, Figure 4). Time to actual visit was on average 57.6 days longer for patients in Group B compared to patients in Group A (*p* = 0.02), averaging at 73.8 days for patients seeking care at the safety net facility (Table 5, Figure 4).

## Discussion

ESAS is increasingly used internationally with attention to defining symptom clusters and potential modulators (39). This study aimed at identifying both the scope of symptom burden in this South Texas population and barriers to obtaining symptom treatment in a palliative care setting. The data suggests a significant symptom burden in oncology patients in this predominantly Hispanic population with over 40% of cancer patients presenting with at least one severe symptom score on initial presentation which is similar to symptom severity in prior studies utilizing ESAS (40). Despite the high symptom burden of these cancer patients, <5% of patients were referred to supportive care clinics. This shows a striking underutilization of symptom burden treatment services despite the demonstrated need. Furthermore, when evaluating patients' symptom score on follow-up visits with their oncologist, there was no statistically significant differences in physical or emotional symptom sub-scores as well as total symptom burden. These symptoms may not be fully addressed during the medical oncology visit highlighting the significant opportunity for supportive care consultation assistance. This may suggest an underlying cultural or educational barrier in oncology clinical practices for the utilization of supportive care services.

Of the symptoms evaluated, pain, tiredness, wellbeing and “other” were most likely to be reported as moderate or severe. Concerns regarding the accuracy of the response to the question of wellbeing will be discussed in our limitations section. Nausea was the least likely to be reported as moderate or severe. However, all symptoms assessed showed both moderate and severe scores without significant outliers. Due to the limited number of patients referred to palliative care, improvement in the ESAS scores of patients who received palliative services vs. those that did not is difficult to evaluate with these data. This study highlights the significant improvements needed to better incorporate palliative care or supportive care into the cancer care continuum. Symptom burden is not routinely assessed in a standardized manner and palliative care continues to be significantly underutilized despite the substantial benefit to both quality of life and survivability for cancer patients. This study identifies the possibility of using a symptom assessment tool and patient reported outcomes to improve the quality of and the access to supportive cancer care. Simple triage tools such as the ESAS form if implemented widely could also offer better insight into symptom burden and cancer disparities across regions and diverse populations.

The study aimed to identify differences in symptom burden among Hispanics vs. non-Hispanic subgroups because of the unique patient population in South Texas. Symptom burden and mean ESAS scores were similar among the two groups without any significant outliers. Except for depression, Hispanics reported slightly less moderate and severe symptom burden across the different measures than non-Hispanics. Considering Hispanic patients with cancer tend to present at later stages of disease (12), it is surprising that the symptom burden seems relatively lower. However, Hispanic cancer patients tend to present at younger ages which may play a role in these findings (12).

In addition, this NCI designated cancer center treats county patients who are uninsured or underinsured through a payer program or safety net program. Symptom burden and access to supportive care services were assessed by comparing insured patients (group A) against the safety-net patients (Group B) to see if insurance status played a role. The two groups were similar in terms of symptom burden severity and frequency but there was a statistically significant difference in patients' access to supportive care services. This investigation revealed that safety-net patients experience a 4-fold delay in the time to the first scheduled palliative care visit when compared to insured patients despite the similar symptom burden profile. These data show a significant disparity to safety-net program patients in access to supportive care services. Barriers to care will need to be further evaluated through future studies.

### Limitations

Though the overall population of cancer patients is adequate for symptom burden review, this study is limited in its evaluation of the palliative care referral patient population by a small sample size and relatively short observation period of 5 months.

In addition, this study found that patients were often confused by the “Well-being” assessment scale frequently scoring a “10” indicating the worst possible being despite all other symptom assessments showing no (0) symptom burden. For this reason, additional patient and staff education regarding clarification of this question are needed in the future.

In the analysis of this data, initial visit was defined as the first ESAS completed. However, this does not necessarily mean that the patient was new to the cancer center. In subsequent studies it will be important to evaluate new patients' initial ESAS scores to follow-up scores. It is possible that we did not capture symptom improvement from initial to follow-up as the majority of patients were actually established patients who may have had symptoms previously addressed by their oncology providers.

This study focused primarily on patient reported outcomes across many types of cancers, and cancer severity was scored per patients' perception of symptom burden. Staging information was not collected in this pilot study, but future studies should aim to include cancer staging in the analysis.

Finally, collection of ESAS scores was dependent on providers returning forms to designated areas for data entry. Therefore it is possible that our data does not reflect all ESAS forms completed in the designated clinics selected for the pilot during the study period. Future standardization of symptom assessment clinical practice and direct entry of data into the electronic medical record would facilitate capturing this patient reported information for all patients. Additionally, with utilization of the electronic record, there are considerable opportunities for designing and implementing provider alerts.

### Future directions

While the ESAS is an excellent tool for evaluating patient distress it may not fully capture all possible distressers as it is limited to only nine categories. The flexibility of the “other” category remains an important element to patient reported outcomes. As financial and spiritual distress has become a priority for many institutions, inclusion of these categories in tools like the ESAS would be beneficial in the future.

The high symptom burden identified at both initial and follow-up visits may indicate an opportunity to develop improved primary palliative care support programs among oncology care teams. Additional education of care teams including nursing would significantly increase the capacity of cancer centers to provide basic or primary palliative care especially for patient populations with demonstrated disparities in access to care. By creating a culture of palliative awareness for cancer care teams these disparities and barriers will likely decrease and facilitate earlier referral to specialized palliative services when indicated.

In addition, expanding this tool to a national level may help better inform healthcare agencies to areas of disparities in symptom burden and in access to quality cancer care.

## Conclusions

This pilot project highlights the high symptom burden of oncology patients and disparities in access to services based on insurance coverage in our South Texas catchment area. This investigation revealed a 4-fold increase in the time to the first scheduled palliative care visit based on whether patients were insured vs. under-insured. While this study is limited by a small sample size, data suggest that under-insured oncology patients in South Texas may have significant barriers to palliative care services, which may influence their cancer care quality.

## Ethics statement

This single center retrospective study was approved by the Institutional Review Board at University of Texas Health Science Center San Antonio (UTHSCSA). This study was granted exemption from human subject consent by the Institutional Review Board at University of Texas Health Science Center San Antonio (UTHSCSA).

## Author contributions

Conception and design: SC and LT; Collection and assembly of data: SC, LT, IA, JJ, and NA; Data analysis and interpretation: SC, LT, and SS; Manuscript writing: SC, LT, SS, and SS-C; Final approval of manuscript: all authors; Accountable for all aspects of the work: all authors.

### Conflict of interest statement

LT Consulting or Advisory Role: Community First Health Plan, Bayer HealthCare Pharmaceuticals. The remaining authors declare that the research was conducted in the absence of any commercial or financial relationships that could be construed as a potential conflict of interest.
