# Supplementary material for: Symptom Burden and Palliative Referral Disparities in an Ambulatory South Texas Cancer Center
Source: Front Oncol. 2018 Oct 15;8:443. doi: 10.3389/fonc.2018.00443 (PMC6196262; doi:10.3389/fonc.2018.00443)
Supplement: Supplementary file 1 [file Data_Sheet_1.PDF]

Appendix 1: Edmonton Symptom Assessment System Revised Version

Edmonton Symptom Assessment System:
(revised version) (ESAS-R)

Please circle the number that best describes how you feel NOW:

No Pain	0	1	2	3	4	5	6	7	8	9	10	Worst Possible Pain
No Tiredness *(Tiredness = lack of energy)*	0	1	2	3	4	5	6	7	8	9	10	Worst Possible Tiredness
No Drowsiness *(Drowsiness = feeling sleepy)*	0	1	2	3	4	5	6	7	8	9	10	Worst Possible Drowsiness
No Nausea	0	1	2	3	4	5	6	7	8	9	10	Worst Possible Nausea
No Lack of Appetite	0	1	2	3	4	5	6	7	8	9	10	Worst Possible Lack of Appetite
No Shortness of Breath	0	1	2	3	4	5	6	7	8	9	10	Worst Possible Shortness of Breath
No Depression *(Depression = feeling sad)*	0	1	2	3	4	5	6	7	8	9	10	Worst Possible Depression
No Anxiety *(Anxiety = feeling nervous)*	0	1	2	3	4	5	6	7	8	9	10	Worst Possible Anxiety
Best Wellbeing *(Wellbeing = how you feel overall)*	0	1	2	3	4	5	6	7	8	9	10	Worst Possible Wellbeing
No __________ Other Problem *(for example constipation)*	0	1	2	3	4	5	6	7	8	9	10	Worst Possible __________
